# 40. Lenzilumab Efficacy and Safety in Newly Hospitalized COVID-19 Subjects: Results From a Phase 3 Randomized Double-Blind Placebo-Controlled Trial

**DOI:** 10.1093/ofid/ofab466.040

**Published:** 2021-12-04

**Authors:** Zelalem Temesgem, Charles Burger, Jason Baker, Christopher Polk, Claudia R Libertin, Colleen F Kelley, Vincent Marconi, Robert Orenstein, Victoria Catterson, William Aronstein, Cameron Durrant, Dale Chappell, Omar Ahmed, Gabrielle Chappell, Andrew Badley, for the LIVE-AIR Study Group

**Affiliations:** 1 Mayo Clinic, Rochester, MN; 2 Hennepin Healthcare Research Institute, Minneapolis, Minnesota; 3 Atrium Health, Charlotte, North Carolina; 4 Mayo Clinic, Jacksonville, Jacksonville, FL; 5 Emory University, Decatur, GA; 6 Atlanta VA, Atlanta, GA; 7 Mayo Clinic in Arizona, Phoenix, AZ; 8 BioSymetrics, Inc, New York, New York; 9 CTI, Clinical Trial Services, Inc, Covington, Kentucky; 10 Humanigen, Inc, Burlingame, California

## Abstract

**Background:**

Severe coronavirus disease 2019 (COVID-19) often results from the immune-mediated cytokine storm, triggered by granulocyte macrophage-colony stimulating factor (GM-CSF), potentially leading to respiratory failure and death. Lenzilumab, a novel anti-human GM-CSF monoclonal antibody, neutralizes GM-CSF and demonstrated potential to improve clinical outcomes in a matched case-cohort study of patients with severe COVID-19 pneumonia. This Phase 3 randomized, double-blind, placebo-controlled trial investigated the efficacy and safety of lenzilumab to improve the likelihood of survival without invasive mechanical ventilation (SWOV), beyond available treatments.

**Methods:**

Hypoxic patients, hospitalized with COVID-19 (n=520), requiring supplemental oxygen, but not invasive mechanical ventilation, were randomized on Day 0 to receive lenzilumab (1800mg, n=261) or placebo (n=259), and available treatments, including remdesivir and/or corticosteroids; and were followed through Day 28.

**Results:**

Baseline demographics were comparable between groups: male, 64.7%; mean age, 60.5 years; median CRP, 79.0 mg/L. Patients across both groups received steroids (93.7%), remdesivir (72.4%), or both (69.1%). Lenzilumab improved the primary endpoint, likelihood of SWOV in the mITT population, by 1.54-fold (HR: 1.54; 95%CI: 1.02-2.32, p=0.0403). Lenzilumab improved SWOV by 1.91-fold (nominal p=0.0073) and 1.92-fold (nominal p=0.0067) in patients receiving remdesivir or remdesivir and corticosteroids, respectively. A key secondary endpoint of incidence of IMV, ECMO or death was also improved in patients receiving remdesivir (p=0.020) or remdesivir and corticosteroids (p=0.0180). Treatment-emergent serious adverse events were similar across both groups.

**Conclusion:**

Lenzilumab significantly improved SWOV in hypoxic COVID-19 patients upon hospitalization, with the greatest benefit observed in patients receiving treatment with remdesivir and corticosteroids. NCT04351152

**Disclosures:**

**Zelalem Temesgem, MD**, **Humanigen, Inc** (Grant/Research Support) **Jason Baker, MD**, **Humanigen, Inc** (Grant/Research Support) **Christopher Polk, MD**, **Atea** (Research Grant or Support)**Gilead** (Advisor or Review Panel member, Research Grant or Support)**Humanigen** (Research Grant or Support)**Regeneron** (Research Grant or Support) **Claudia R. Libertin, MD**, **Gilead** (Grant/Research Support) **Colleen F. Kelley, MD, MPH**, Gilead Sciences (Individual(s) Involved: Self): Grant/Research Support; Moderna (Individual(s) Involved: Self): Grant/Research Support; Novavax (Individual(s) Involved: Self): Grant/Research Support; Viiv (Individual(s) Involved: Self): Grant/Research Support **Vincent Marconi, MD**, **Bayer** (Consultant, Scientific Research Study Investigator)**Eli Lilly** (Consultant, Scientific Research Study Investigator)**Gilead Sciences** (Consultant, Scientific Research Study Investigator)**ViiV** (Consultant, Scientific Research Study Investigator) **Victoria Catterson, PhD**, **Humanigen, Inc** (Consultant) **William Aronstein, MD, PhD**, **Humanigen, Inc** (Consultant) **Cameron Durrant, MD**, **Humanigen, Inc** (Employee) **Dale Chappell, MD**, **Humanigen, Inc** (Employee) **Omar Ahmed, PharmD**, **Humanigen, Inc** (Employee) **Gabrielle Chappell, MSc**, **Humanigen, Inc** (Consultant) **Andrew Badley, M.D.**, **AbbVie** (Consultant) **for the LIVE-AIR Study Group, n/a**, **Humanigen, Inc** (Grant/Research Support)

